# 626. Tebipenem Pharmacokinetics and Soft-Tissue Distribution in Diabetic Patients with Lower Extremity Infections using *In Vivo* Microdialysis

**DOI:** 10.1093/ofid/ofac492.678

**Published:** 2022-12-15

**Authors:** Yasmeen Abouelhassan, Andrew J Fratoni, Ashley Shepard, David P Nicolau, Tomefa E Asempa

**Affiliations:** Hartford Hospital, Hartford, Connecticut; Hartford Hospital, Hartford, Connecticut; Hartford Hospital, Hartford, Connecticut; Hartford Hospital, Hartford, Connecticut; Hartford Hospital, Hartford, Connecticut

## Abstract

**Background:**

Administration of the oral carbapenem, tebipenem along with its broad spectrum of activity against anaerobic, Gram-positive and Gram-negative pathogens including extended-spectrum β-lactamase-producing Enterobacterales, offers clinicians a potential new option and route to treat a range of infections. Lower extremity infections in diabetic patients are associated with high rates of hospitalization and amputation. Given the microvascular dysfunction and poor peripheral circulation in this population, the aim of this study was to assess tebipenem soft tissue pharmacokinetics (PK) and interstitial fluid distribution among diabetic patients with lower extremity infections using *in vivo* microdialysis.

**Methods:**

This was a single-center, open-label, observational PK study in diabetic patients with foot infections who were enrolled and received tebipenem (600 mg) orally q8 hrs for a total of 3 doses. A microdialysis catheter was inserted within 4-8 cm of the wound margin to allow for dialysate sampling. Ten concurrent plasma and dialysate samples over an 8 hr period starting immediately prior to the last dose of tebipenem were obtained. Protein binding was determined by ultracentrifugation at 1 hr post-third dose. Plasma and dialysate samples were assayed via a validated LC/MS/MS assay. Non-compartmental analyses for free plasma and soft-tissue concentration were used to obtain PK parameters.

**Results:**

Four male diabetic patients with an age of 70 ± 7 years and a hemoglobin A1C of 10 ± 2% were consented. All patients had an active complicated skin and soft tissue infection as defined by PEDIS Grade 2 or 3. Mean ± standard deviation (SD) plasma protein binding was 50% ± 1%. Mean ± SD tebipenem PK parameters in plasma were: maximum free concentration (*f*C_max_), 2.34 ± 0.94 mg/L; time to C_max_ (T_max_), 2.99 ± 1.40 hr; half-life (t_1/2_), 2.23 ± 1.65 hr and free area under the concentration-time curve (*f*AUC*_p_*_(0-8)_): 8.37 ± 1.63 mg.h/L. Mean ± SD parameters in tissue were: C_max,_ 2.50 ± 1.04 mg/L; T_max,_ 2.99 ± 1.40 h; t_1/2_ 1.99 ± 1.42 hr; and AUC*_t_*_(0-8)_, 8.01 ± 0.76 mg.h/L.

**Conclusion:**

This study demonstrates that tebipenem has excellent distribution into interstitial fluid and lower extremity tissue of diabetic patients with ongoing foot infections.

**Disclosures:**

**Yasmeen Abouelhassan, PhD**, Pfizer: spouse salary **David P. Nicolau, PharmD**, Shionogi: Grant/Research Support.

